# Predicting antibiotic resistance in complex protein targets using alchemical free energy methods

**DOI:** 10.1002/jcc.26979

**Published:** 2022-08-25

**Authors:** Alice E. Brankin, Philip W. Fowler

**Affiliations:** ^1^ Nuffield Department of Medicine, John Radcliffe Hospital University of Oxford Oxford UK; ^2^ National Institute of Health Research Oxford Biomedical Research Centre John Radcliffe Hospital Oxford UK

**Keywords:** alchemical free energy methods, antibiotic resistance, molecular dynamics, relative binding free energy calculations, tuberculosis

## Abstract

Drug resistant *Mycobacterium tuberculosis*, which mostly results from single nucleotide polymorphisms in antibiotic target genes, poses a major threat to tuberculosis treatment outcomes. Relative binding free energy (RBFE) calculations can rapidly predict the effects of mutations, but this approach has not been tested on large, complex proteins. We use RBFE calculations to predict the effects of *M. tuberculosis* RNA polymerase and DNA gyrase mutations on rifampicin and moxifloxacin susceptibility respectively. These mutations encompass a range of amino acid substitutions with known effects and include large steric perturbations and charged moieties. We find that moderate numbers (*n =* 3–15) of short RBFE calculations can predict resistance in cases where the mutation results in a large change in the binding free energy. We show that the method lacks discrimination in cases with either a small change in energy or that involve charged amino acids, and we investigate how these calculation errors may be decreased.

## Graphical abstract

Relative binding free energy (RBFE) calculations pose a potential avenue to predict antibiotic resistance associated with single nucleotide polymorphisms. We investigated whether RBFE calculations could be applied to two large, complex and clinically important protein targets in *Mycobacterium tuberculosis*. We show that RBFE calculations can predict resistance in cases where the mutation results in a large change in the binding free energy and investigate the sources of error for more complex mutations.

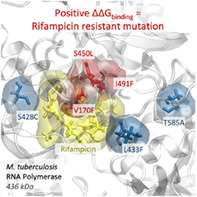

## 1 INTRODUCTION

Tuberculosis is a difficult disease to treat; the standard regimen is four antibiotics, rifampicin, isoniazid, pyrazinamide, and ethambutol, for 6 months. An infection that is resistant to both rifampicin and isoniazid is called multi‐drug resistant tuberculosis (MDR‐TB) and the treatment regimen recommended by the World Health Organization (WHO) is complex but always includes levofloxacin or moxifloxacin, which are fluoroquinolones.^1^

**FIGURE 1:**
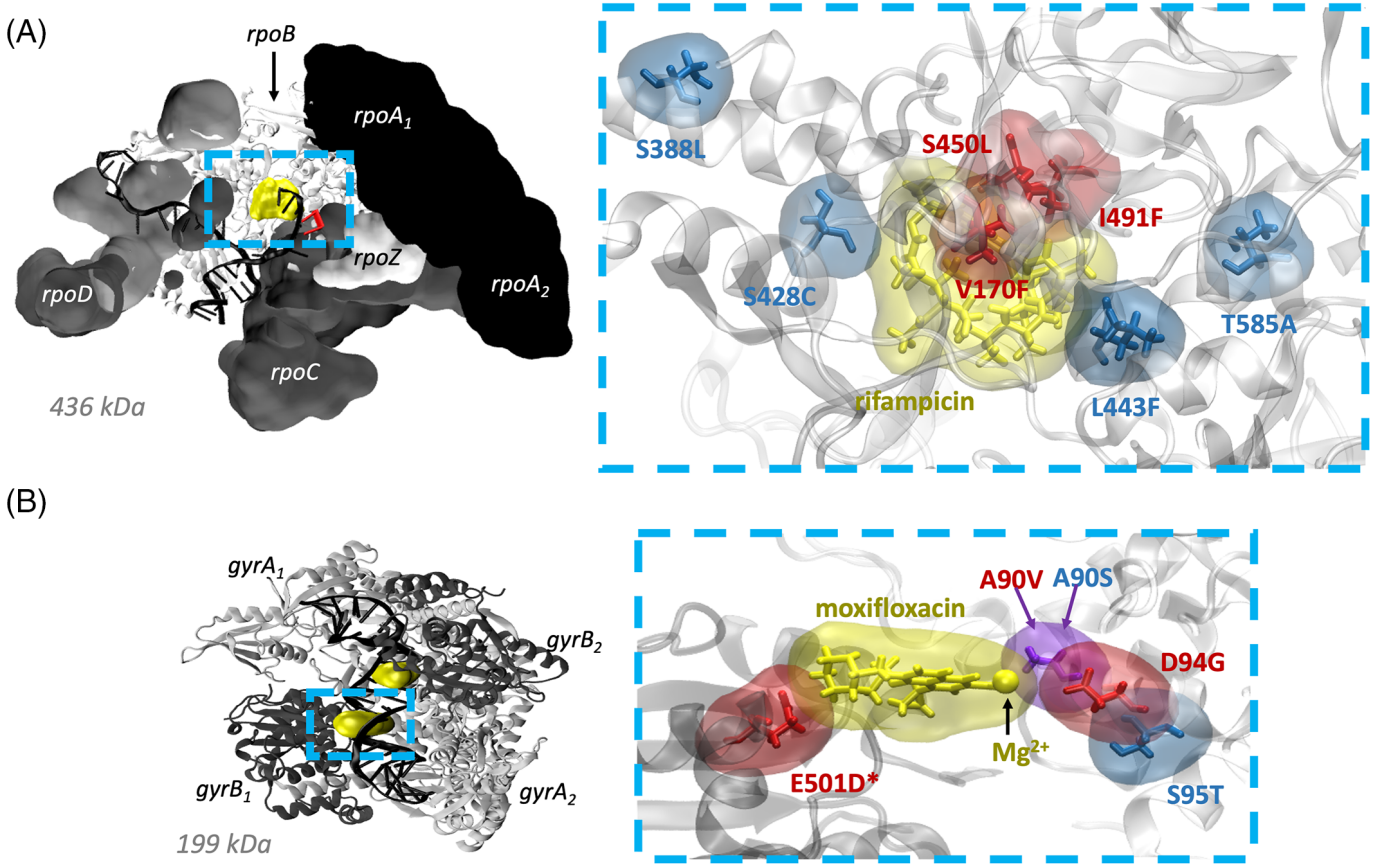
Structures of *Mycobacterium tuberculosis* (A) RNA polymerase (RNAP)^28^ and (B) DNA gyrase (DNAG)^27^ cleavage complex, showing the selected clinical mutations associated with antibiotic resistance and susceptibility relative to the antibiotic binding sites. For clarity, RNAP subunits (excluding rpoB) are shown in surface view and nucleic acids are hidden in close‐up visualizations. Resistance‐conferring mutations are drawn in red, those associated with susceptibility blue and those residues where different mutations confer different resistance phenotypes purple. An asterisk (*) indicates a *gyrB* mutation.

Rifampicin (RIF) acts by binding to the β‐subunit of the RNA polymerase (RNAP, encoded by the *rpoB* gene), preventing the extension of the RNA (Figure 1A). The most common resistance‐conferring mutation is *rpoB* S450L, however a wide range of mutations have been observed clinically.^2^, ^3^, ^4^, ^5^ The majority of these are found in amino acids 428 to 452 which pack against the drug (usually known as the “rifampicin resistance determining region” or RRDR), enabling the development of nucleic acid amplification tests, such as the Cepheid GeneXpert MTB/RIF system which is endorsed by the WHO for diagnosis of MDR‐TB.^6^, ^7^ Not all non‐synonymous mutations in the RRDR, however, confer resistance, for example *rpoB* L443F.^5^ Nor does resistance arise purely within the RRDR: *rpoB* I491F and V170F are proximal to S450L and the former was suspected to be behind an outbreak of MDR‐TB in Eswatini since it is not detected by GeneXpert.^8^

The fluoroquinolones target the DNA gyrase (DNAG), a tetrameric enzyme which unwinds DNA by forming and re‐ligating double stranded DNA breaks prior to transcription and replication (Figure 1B). Specifically, two fluoroquinolone molecules intercalate into DNA breaks and bind specific *gyrA* residues via a coordinated Mg^2+^ ion. This stabilizes DNA–DNA gyrase covalent linkages and prevents re‐ligation of DNA double stranded breaks.

The most common DNA gyrase mutations found in MDR‐TB samples are *gyrA* D94G and *gyrA* A90V and these mutations are strongly associated with fluoroquinolone resistance.^2^, ^3^, ^9^, ^10^, ^11^, ^12^ These residues are part of the *gyrA* “quinolone resistance determining region” (QRDR), defined as *gyrA* codons 74–113.^13^ However, again, not all mutations in this region confer resistance, leading to false positive resistance results in genotypic assays.^14^ Rarely seen DNA gyrase mutations in *gyrB* are also associated with fluoroquinolone resistance, and a *gyrB* QRDR from residues 461 to 501 has also been proposed.^15^ The residues of the two QRDR regions make up the fluoroquinolone binding pocket, and *gyrB* A642P is the only mutation significantly associated with an increase in minimum inhibitory concentration (MIC) to fluoroquinolones that was found outside this region.^9^

**FIGURE 2:**
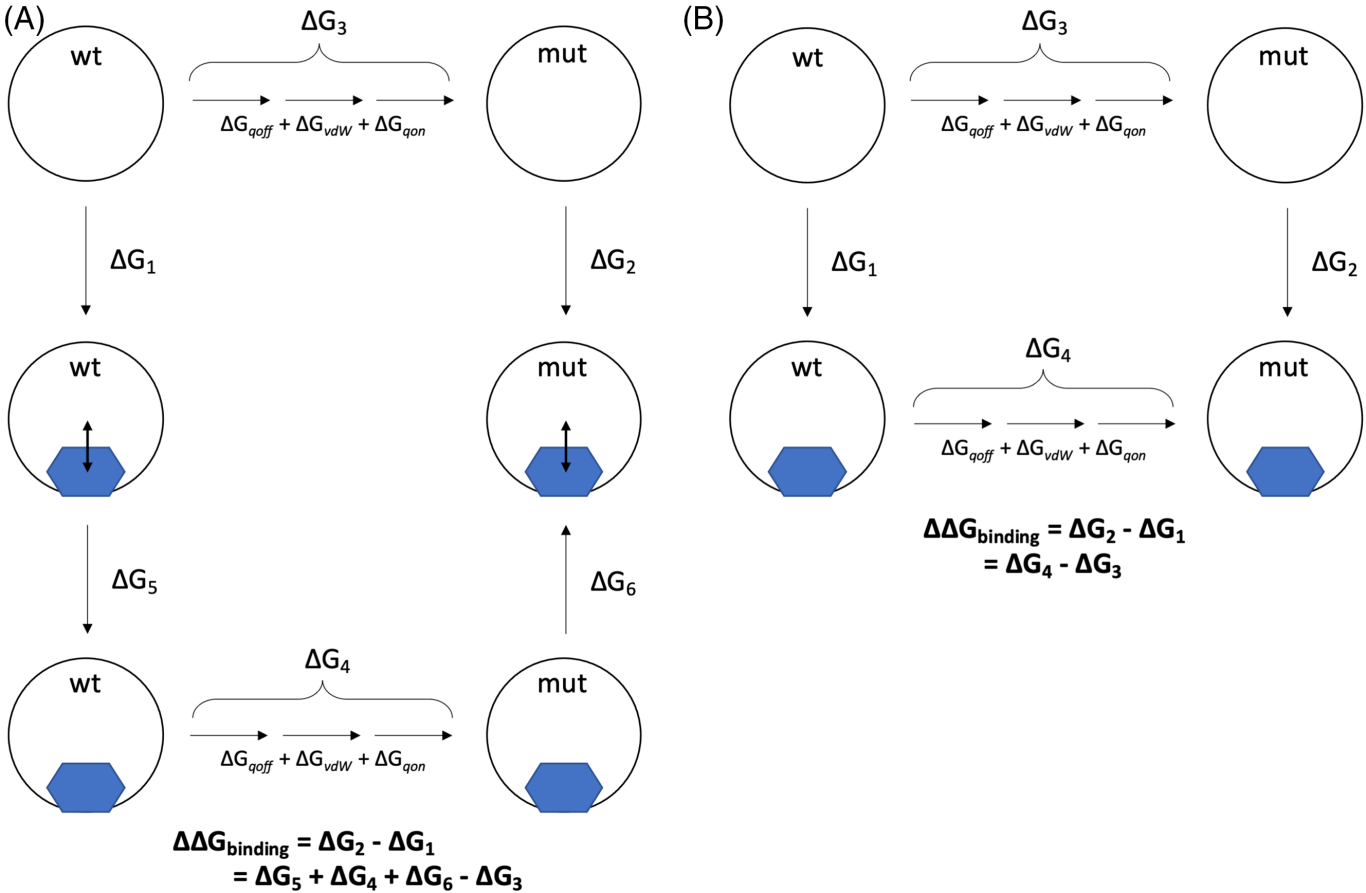
Free energy cycles for (A) rifampicin binding RNAP and (B) moxifloxacin binding DNAG gyrase cleavage complex. The subscripts _qoff_, _vdW_, and _qon_ describe the process of first removing the electrical charge from atoms being perturbed, followed by transforming their van der Waals parameter, before finally recharging the atoms being perturbed. Double headed arrows represent the restraint used to prevent rifampicin from leaving the binding pocket. In all cases we are making use of the fact that free energy is a state function and therefore we can write the difference binding free energy (ΔΔ*G*_binding_) as a sum of so‐called alchemical free energies (e.g., Δ*G*_4_–Δ*G*_3_)

We assume that mutations cause resistance by reducing the affinity of an antibiotic ligand for its target. Since we are only interested in whether a mutation increases or decreases the antibiotic's affinity for the target, the difference in binding free energy (ΔΔ*G*) between the wild type and mutant systems is calculated. This can be achieved by employing relative binding free energy (RBFE) methods, whereby a wild type amino acid is transmuted into the mutant along a non‐physical pathway defined by a progress coordinate, 0 ≤ *λ* ≤ 1. For equilibrium‐based methods, a series of short molecular dynamics (MD) simulations are performed at fixed values of λ and the resulting Δ*G* values are related to the difference in binding free energy via a thermodynamic cycle (Figure 2).

RBFE approaches have so far been successfully applied in small molecule drug design where the effect of perturbations to a lead compound on the binding affinity to its target can be predicted to within 1 kcal mol^−1^ error.^16^, ^17^, ^18^ The regular nature of amino‐acids compared to small molecules means that forcefields are well parameterized for amino acids^19^ but potentially less so for small drug‐like molecules^20^ and therefore applying RBFE to predict the effects of amino acid mutations may be more accurate. Indeed, RBFE methods have been shown to yield accurate resistance predictions for genetic mutations associated with disease.^21^, ^22^, ^23^, ^24^ However, these studies have focused on small, monomeric protein targets, for example, we previously used RBFE methods to successfully predict trimethoprim resistance associated with mutations in the *Staphylococcus aureus* dihydrofolate reductase protein, which comprises 157 residues.^21^, ^25^

In this paper, to assess how well the method can be applied to much larger systems, we shall apply the same approach to two large protein complexes, the RNA polymerase (4671 residues) and the DNA gyrase cleavage complex (1473 residues), to assess how well we can predict the effect of seven and five mutations on the action of rifampicin and moxifloxacin, respectively. We emphasize that we define success as the ability of the method to rapidly predict whether each mutation confers resistance or not to the relevant drug. This qualitative approach is different to most RBFE studies which instead assess how well the method can calculate the quantitative change in binding free energy (which are unknown for these proteins in any case). To retain the ability for the method to rapidly return results in any future implementation, we have used standard parameters (e.g., for ions), even if this potentially reduces our accuracy or precision.

### 1.1 Selecting the mutations studied

To test the ability of RBFE to predict antibiotic resistance we selected a small number of mutations in the RNAP and DNAG that confer resistance; to act as negative controls we added several more mutations known to have no clinical effect. We chose to test the most common resistance‐conferring mutations for each drug. For RNAP this is S450L in the RRDR of *rpoB* and for the DNA gyrase these are A90V and D94G in the QRDR of *gyrA* (Figure 1A,B). *gyrA* D94G is a robust test of RBFE methods as the mutation involves a significant change in amino acid properties and electrical charge. For rifampicin we also selected V170F and I491F in *rpoB* which both confer resistance, are proximal to both S450L and the antibiotic binding site, but are not in the RRDR (Table 1, Figure 1A). *rpoB* I491F is one of the so‐called “disputed” mutations which either have variable or borderline rifampicin minimum inhibitory concentrations (MICs).^12^, ^26^ For moxifloxacin we also tested E501D in *gyrB* ^12^ which is close to the antibiotic binding site but not in the *gyrA* QRDR (Table 1, Figure 1B).

**TABLE 1:** Summary of distances of RNAP and DNAG mutations from the drug binding site.

Protein	Mutation	Expected result	Distance from drug (Å)
RNAP	S388L	Susceptible to rifampicin	9.0
	S428C	Susceptible	4.0
	L443F	Susceptible	10.4
	T585A	Susceptible	12.3
	V170F	Resistant	4.5
	S450L	Resistant	2.6
	I491F	Resistant	3.4
DNAG	S95T	Susceptible to moxifloxacin	9.0
	A90S	Hyper‐susceptible	2.7
	A90V	Resistant	2.7
	E501D^a^	Resistant	2.6
	D94G	Resistant	5.1

*Note*: Measurements are taken as the minimum distance between the wild‐type amino acid and the drug. For DNAG, where there are two gyrA and gyrB proteins and two moxifloxacin molecules bound, measurements are taken for the wild‐type amino acid in the A or B chain (for *gyrA* and *gyrB*, respectively) to the nearest bound moxifloxacin molecule.

^a^ Indicates a *gyrB* mutation.

When choosing negative controls, we prioritized mutations that were observed multiple times in clinical samples, are close to the drug binding site and do not involve a charge change or a proline residue. For the RNAP, L443F was selected since it lies within the RRDR and is close to the rifampicin binding site yet does not confer resistance^11^ and therefore is a good negative control (Table 1, Figure 1A). We also selected S388L and T585A which are further from the binding site and are seen in clinical samples. Finally, we chose an amino acid (Ser428) at which non‐synonymous mutations are expected to confer resistance, since it lies in the RRDR, but for which no firm statistical association has yet been made, and chose a mutation (S428C) which minimally chemically perturbs the sidechain. We expect this to not confer resistance, since it has not been observed clinically and the sidechain points away from the drug and S428C is therefore a good, if somewhat artificial, negative control.

For the DNA gyrase negative controls, we chose *gyrA* S95T (Figure 1B) since it is very common—it is found in almost all samples except the H37Rv reference genome—and is within the QRDR. Testing different mutations at the same position which have different effects is a particularly stringent test of the ability of RBFE methods to predict antibiotic resistance. We therefore also tested the *gyrA* A90S mutation (Figure 1B) – this is not seen clinically but a serine is present at the equivalent position in the DNA gyrase of other bacterial species and is suggested to help stabilize the gyrase‐fluoroquinolone complex via participation in water‐ion bridging interactions with the drug coordinated Mg^2+^. *Mycobacterium tuberculosis* has some innate immunity to fluoroquinolones which has been suggested is due to the alanine at this position.^27^ The *gyrA* A90S mutation is therefore expected to strengthen the binding of moxifloxacin, thereby conferring hyper‐susceptibility.

## 2 METHODS

### 2.1 RNA polymerase and DNA gyrase system setup

The structure of the *M. tuberculosi*s RNA polymerase (PDB: 5UH6),^28^ including a 14‐base stretch of DNA, 2 RNA nucleotides, two zinc ions, a magnesium ion and a bound rifampicin molecule, was solvated with 114,838 waters and 127 sodium ions—the latter to ensure electrical neutrality—creating a cubic simulation unit cell of initial dimensions 20.1 × 15.2 × 13.1 nm^3^. The flexible loop region of each *gyrB* protein that were not resolved in the structure of the *M. tuberculosis* DNA gyrase cleavage complex (PDB:5BS8)^27^ were modeled in using the ModLoop server.^29^ This structure, including the 19‐base stretch of DNA, four Mg^2+^ ions, two bound moxifloxacin molecules and 403 crystal waters was placed in a rhombic dodecahedron unit cell with dimensions 13.8 × 13.8 × 9.8 × 0.0 × 0.0 × 0.0 × 0.0 × 6.9 × 6.9 nm^9^. The unit cell was solvated with 59,895 waters, 175 Na^+^ and 112 Cl^−^ ions providing electrical neutrality and a 100 mM salt concentration. The generalized AMBER and AMBER ff99SB‐idln forcefields were used to parameterize all protein chains, nucleic acids, ligands and ions.^30^ To facilitate the covalent bond between *gyrA* Tyr129 and the phosphate backbone of DNA by GROMACS, two modified amino acids (TYX and TYY) were created. These “hybrid residues” contained the parameters for Tyr, excluding the hydroxyl hydrogen, all nucleotides in the covalently bound DNA chain and the covalent bond between the Tyr hydroxyl oxygen and the corresponding DNA backbone phosphorus atom. The PDB file order and residue naming was adjusted to reflect the modified amino acids. Due to the size of the hybrid residues (403 atoms) it was not possible to redistribute the partial charges in a principled way using QM/MM and we instead choose to simply retain the partial charges of DNA and Tyr as found in the forcefield. Due to the “loss” of the hydrogen atom from the tyrosine the system was left with a non‐integer charge, so a solvent chloride ion was modified to provide a balancing charge. This is clearly not optimal, however we felt it preferable to manually redistributing charge.

The energies of the resulting RNA polymerase and DNA gyrase unit cells of 396,776 atoms and 205,883 atoms, respectively, were then minimized by GROMACS^31^ 2016.3 and 2018.2 respectively, using a steepest descent algorithm for 1000 steps before being gradually warmed from 100 to 310 K over 500 ps. For comparison, the DHFR unit cell only contained 27,115 atoms.^21^, ^25^ The drug was removed from the DNAG structure creating a presumed apo state; the resulting apo and complexed systems were each equilibrated for 5 × 50 ns. By contrast only three drug bound RNAP 50 ns simulations were run, from which the drug was subsequently removed as required to create presumed apo structures. The DNAG apo structures used to seed the alchemical simulations are therefore much more likely to be equilibrated than the apo RNAP structures. The temperature was maintained at 310 K using a Langevin thermostat with a time constant of 2 ps. An isotropic Parrinello‐Rahman barostat with a 1 ps time constant and a compressibility of 4.46 × 10^−5^ bar^−1^ was applied to keep the pressure at 1 bar. Electrostatic forces were calculated using the particle mesh Ewald algorithm with a real space cutoff of 1.2 nm whilst van der Waals forces were only calculated between atoms less than 1.2 nm apart with a switching function applied from 0.9 nm. The lengths of all bonds involving a hydrogen were constrained using LINCS,^32^ permitting a timestep of 2 fs. For DNA gyrase, to prevent the moxifloxacin coordinated Mg^2+^ from dissociating from moxifloxacin, we used a harmonic distance restraint of sufficient strength (100,000 kJ mol^−1^ nm^−2^) to maintain the distance observed in the crystal structure (0.209 nm) throughout all simulations, lower values were not sufficient. A series of assumed independent structures were obtained by saving the coordinates of the system every 10 ns from each of three RNAP equilibration simulations and from each of the five DNAG equilibration simulations.

Mutations were then introduced into each of these structures using pmx.^33^ To reduce the likelihood of clashes between the “new” sidechain and the remainder of the protein (i.e., in simulations with *λ* ~ 1) we then applied a short Alchembed procedure^34^ to each structure – this involved a 1000 step simulation where *λ* was increased from 0 to 1 using a soft‐core van der Waals potential. For the RNAP simulations, we found it necessary to then apply a very short 0.5 ps simulation during which the temperature was increased to 310 K, presumably because this system had not been subjected to same degree of equilibration as DNAG. This created a pool of presumed independent mutated structures that could be used to seed alchemical thermodynamic integration simulations.

Following best practice,^35^ the free energies (Figure 2) required to remove the electrical charge on the perturbing atoms (Δ*G*_qoff_), transmute the van der Waals parameters (Δ*G*_vdW_) and recharge the remaining atoms (Δ*G*_qon_) were separately calculated using GROMACS 2016.3 for RNAP and 2019.1 for DNAG. Each calculation required eight simulations at equally spaced values of the progress parameter, *λ*. To accelerate convergence, 10,000 replica exchanges were attempted between neighboring *λ*‐simulations every 1000 timesteps. To assess convergence, the percentage overlap between neighboring *λ* windows was calculated using the numpy.histogram tool from NumPy. The process was repeated for both apo and complexed forms of either the RNAP or DNAG, thereby resulting in six independent free energies (Figure 2). The timestep was reduced from 2 to 1 fs and LINCS constraints were removed for all vdW transitions and the qon transition of *gyrA* D94G to prevent crashing. To ensure rifampicin remained bound, a harmonic distance‐based potential with spring constant 1000 kJ mol^−1^ nm^−2^ was applied between the centers of mass of the drug and the RNAP beta subunit. Two additional free energies describing the cost of removing this restraint (Figure 2) were then also calculated. In hindsight, rifampicin would likely have remained bound without a restraint – since the restraint was not necessary to keep the drug bound the free energies calculated for removal of the restraint were minimal (supplementary file “rpob_summary.csv”), however, to ensure consistency between repeats we kept the restraint throughout.

Files and scripts necessary to reproduce the above steps, starting with the alchembed step, for the *gyrA* A90V DNAG and *rpoB* S450L RNAP mutations can be found here: https://github.com/fowler-lab/tb-rbfe-setup.

### 2.2 Calculation of errors

In previous studies of *S. aureus* DHFR^21^, ^25^ all alchemical free energy calculations were repeated the same number of times which, since *n* values of the final difference in binding free energy (ΔΔ*G*) were then obtained, simplified the calculation of errors. Both simulation unit cells studied here were over an order of magnitude larger and we therefore instead calculated the *SEM* at the level of each individual alchemical free energy (e.g., Δ*G*_vdW_), with the final error in ΔΔ*G* estimated by adding these in quadrature. Throughout a 95% confidence limit was estimated by multiplying the *SE* by the appropriate t‐statistic. We arbitrarily decided that at least three independent values of each alchemical free energy would be calculated, and then additional repeats would be run with the aim of reducing the magnitude of the overall 95% confidence limit to less than 1 kcal mol^−1^. Achieving the latter was not always possible even when large numbers of repeats were run (*n* ≥ 10, see Supplementary Information S1).

### 2.3 Simulations run

Overall, 241 alchemical free energies, each requiring 8× *λ* simulations 0.5 ns long, were calculated for the RNA polymerase allowing the six mutations to be studied. When the equilibration simulations are included, this is a total of 1.11 μs of MD simulations. To study the five DNA gyrase mutations, a total of 231 alchemical free energies were calculated (8× *λ* simulations 0.5 ns long) and including equilibration simulations, a total of 1.17 μs of MD simulations were initially performed. As described later, for DNA gyrase, nine calculations were extended to 5 ns which increased the total MD performed to 1.49 μs. To avoid equilibration effects, the first 0.25 ns of each *λ* simulation was discarded before measuring the Δ*G* using thermodynamic integration calculated with the trapezoidal rule. Specifically, we used the numpy.trapz tool from NumPy for the integration calculation. Since the decision to discard 250 ps from the start of each simulation was arbitrary, we investigated whether discarding different amounts of data altered the results for one of the simple susceptible mutations, *gyrA* S95T, and the most complex mutation, *gyrA* D94G. Discarding either 125 or 375 ps, compared to 250 ps, led to no significant difference in the final calculated ΔΔG measurement for *gyrA* S95T or *gyrA* D94G (Figure S1).

## 3 RESULTS

### 3.1 Predictions

The simplest approach is to assume that a positive value of the change in binding free energy of the antibiotic (*ΔΔG* > 0) indicates that the antibiotic binds less well to the target following the mutation and therefore would be predicted to confer resistance to that drug. Clinically, however, a sample is categorized as “resistant” if its minimum inhibitory concentration (MIC) is greater than a critical concentration, often the epidemiological cut off value (ECOFF/ECV), which is defined as the MIC of the 99th percentile of a collection of phenotypically‐wildtype samples. Such thresholds for both drugs were derived using published ECOFF/ECV values^36^ as described previously.^21^

Three independent values of ΔΔG were first calculated. Each value of ΔΔ*G* required the calculation of 6–8 alchemical free energies (Figure 1, Methods). Repeats of the alchemical free energy components exhibiting the greatest variation were then run to efficiently reduce the confidence limits of the prediction as described in the Methods. First let us consider the overall values of ΔΔ*G* and whether successful predictions can be made.

**FIGURE 3:**
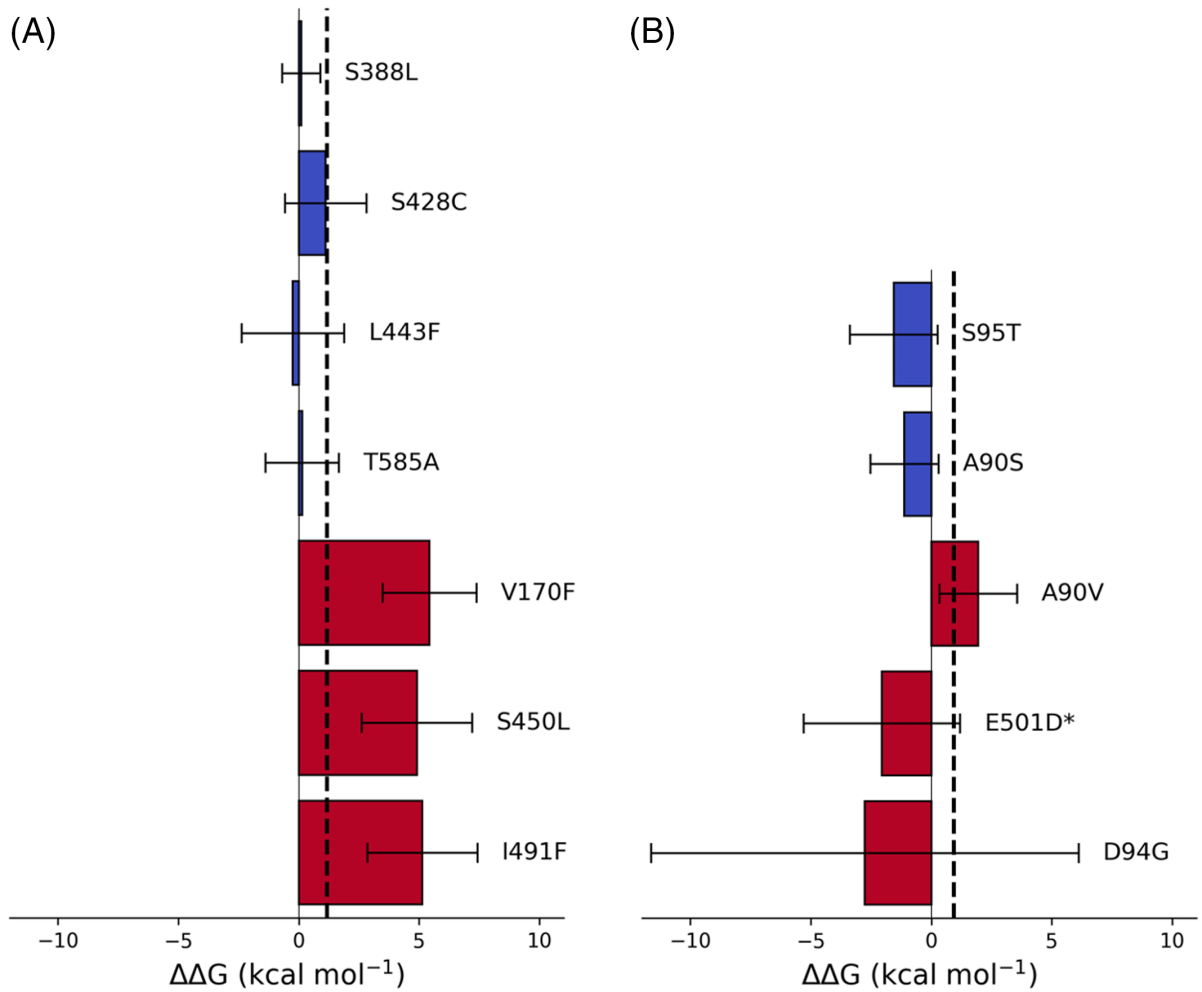
The calculated effect of the listed mutations on the binding free energy of (A) rifampicin to RNAP and (B) moxifloxacin to DNA gyrase. Dotted lines represent the value of ΔΔ*G* equivalent to the epidemiological cutoff value for (A) rifampicin and (B) moxifloxacin; above this value an *Mycobacterium tuberculosis* isolate would be considered clinically resistant. Bars represent the mean ΔΔ*G* for each susceptible (blue) and resistant (red) mutation compared to the wild‐type protein and 95% confidence limits are shown, calculated using the appropriate *t*‐statistic. An asterisk (*) indicates a *gyrB* mutation.

For rifampicin, only one of the four negative controls (S388L) was correctly predicted to have no effect on the action of rifampicin (Figure 3A); since the confidence limits of S428C, L443F, and T585A all bracket the ECOFF threshold no definite prediction could be made for these mutations. Clinically the method as implemented would therefore return an “Unknown” phenotype for these mutations. All three rifampicin‐resistance conferring mutations, including the disputed mutation I491F, not only have positive values of ΔΔ*G* but also lie above the clinical threshold derived from the ECOFF/ECV. These mutations are therefore correctly predicted to confer resistance to rifampicin.

Both moxifloxacin negative controls (*gyrA* S95T and A90S) were correctly predicted to not affect the binding of moxifloxacin to the DNA gyrase (Figure 3B). Although hyper‐susceptibility is expected for A90S, the magnitude of the confidence limits prevents us drawing any conclusions. No definite prediction could be made for any of the three mutations associated with moxifloxacin resistance since the confidence limits of all three mutations straddled the clinical threshold. Unlike the RNA polymerase, two of the mutations to the DNA gyrase involved charged residues (*gyrB* E501D and *gyrA* D94G) and not surprisingly these had the largest estimated errors.

To see how our *ΔΔG* values compared with clinical resistance measurements, we calculated an estimated “expected *ΔΔG*” from the geometric mean of MICs associated with each of the resistance conferring mutations, using previously described methods.^21^ However, the errors in both the “expected ΔΔ*G*” and the ΔΔ*G* values calculated by RBFE were too large to enable us to draw any conclusions about how well the values compare with one another (Figure S2). We also note that the expected free energy for the resistance conferring *rpoB* I491F mutation does not cross the clinical threshold we applied for resistance. Although the mutation is accepted as conferring resistance,^37^ we had few clinical examples of this mutation, several of which had low MICs leading to a low expected ΔΔ*G* value.

### 3.2 Investigation of sources of error

**FIGURE 4:**
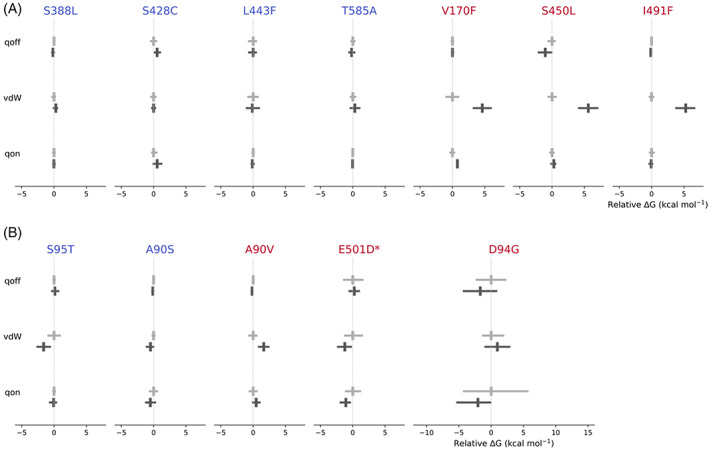
Apo (light gray) and drug‐bound (dark gray) free energy calculations for (A) RNAP and (B) DNA gyrase mutations for de‐charging (qoff), van der Waals (vdW), and re‐charging (qon) transitions. All results are normalized to the mean of the calculations for the apo leg for each transition for each mutation. Mean values are denoted by a cross and the error bars describe the 95% confidence limits, calculated from the *SEM* using the appropriate *t*‐statistic. The free energy cost of removing the restraints for rifampicin is not shown since for all mutations it is negligible, indicating that restraints were likely not required to keep the drug in the binding site. An asterisk (*) indicates a *gyrB* mutation.

The magnitudes of the estimated errors prevented us from making a definite classification in 6 of the 12 mutations studied. To further examine what is driving the magnitudes and confidence limits of the individual *ΔΔG* values in Figure 3, we analyzed the alchemical free energy components from the de‐charging (Δ*G*_qoff_), van der Waals (Δ*G*_vdW_) and re‐charging (Δ*G*_qon_) transitions (Figure 2) for both apo and drug‐bound legs of the free energy calculations (Figure 4). As expected, for both the RNA polymerase and the DNA gyrase, there were no significant differences for the negative control mutations between the mean apo and drug‐bound values of Δ*G*_qoff_, Δ*G*_vdW_, and Δ*G*_qon_ and the estimated error is generally small.

For all three resistance‐conferring mutations in *rpoB* the value of Δ*G*_vdW_ when rifampicin is bound is significantly greater than the same transition for the apo protein and it is this that is mainly driving the positive value of ΔΔ*G*. The difference between the apo‐ and rifampicin‐bound vdW transitions for V170F, S450L, and I491F are 4.6, 5.6, and 5.3 kcal mol^−1^, respectively. Since all three of these mutations involve the introduction of a larger sidechain that is oriented towards the bound drug, this is consistent with resistance arising primarily through steric hindrance of the rifampicin binding site. For comparison, despite a similar number of atoms being perturbed, there was no difference in the apo‐ and drug‐bound values of Δ*G*_vdW_ for the susceptible mutation *rpoB* L443F, which is also in the RRDR (Figure 1A) and, whilst this also involves the introduction of a larger sidechain, in the crystal structure this is directed away from rifampicin. Differences in Δ*G*_vdW_ between apo‐ and complexed DNA gyrase also appear mainly responsible for the positive value of ΔΔ*G* for *gyrA* A90V, however the net effect is reduced.

**FIGURE 5:**
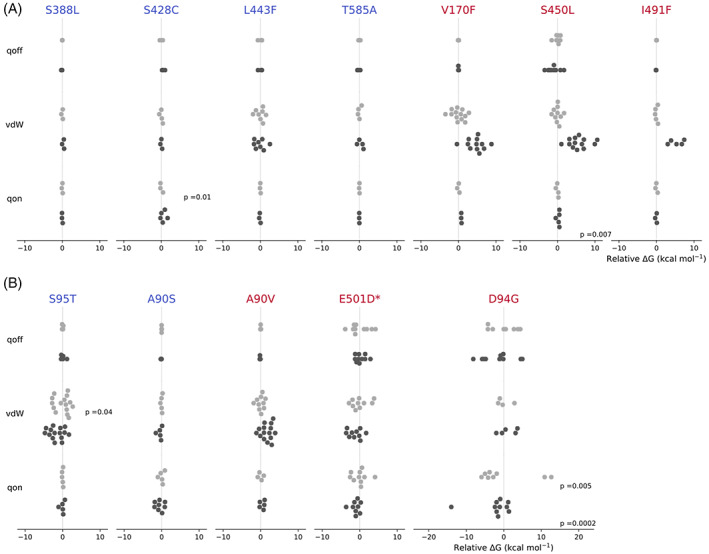
Swarm plots of individual results from apo (light gray) and drug bound (dark gray) alchemical free energy calculations for mutations in the RNA polymerase (A) and DNA gyrase (B). All results are normalized to the mean of the calculations for the apo leg for each qoff, vdW, or qon transition for each mutation. *P*‐values from Shapiro Wilks test are displayed for each transition showing evidence of non‐normality in the repeated calculations, transitions where no *p*‐value is shown indicates there was no evidence of non‐normality in the data (*p* > 0.05). An asterisk (*) indicates a *gyrB* mutation.

Hence the variation in ΔΔ*G* arises mainly from the apo and complexed values of Δ*G*_vdW_ – the notable exceptions being *gyrB* E501D and *gyrA* D94G. This is despite our efforts to minimize the overall error by running up to 4× the number of repeats for those transitions (Table 2) to reduce their individual estimated errors. For *gyrB* E501D and *gyrA* D94G all three transitions contribute significant error, which since they add in quadrature, leads to a large overall error in ΔΔ*G*. This is not surprising since both mutations involve turning off (and on) electrical charge and D94G involves a net charge change that must be compensated for elsewhere in the system. To investigate how far we might reduce the errors, let us now consider the individual values of Δ*G*_qoff_, Δ*G*_vdW_ and Δ*G*_qon_ (Figure 5).

**TABLE 2:** Summary of free energy calculations for RNAP and DNAG mutations.

Protein	Mutation	Expectation	ΔΔ*G* (kcal mol^−1^)	*N* ^a^	*N*_min_ ^b^	*N*_max_ ^b^
RNAP	S388L	Susceptible	0.1 + 0.8	18	3	3
	S428C	Susceptible	1.1 ± 1.7	21	3	5
	L443F	Susceptible	−0.2 ± 2.1	28	3	8
	T585A	Susceptible	0.2 ± 1.5	20	3	4
	V170F	Resistant	5.4 ± 1.9	37	3	13
	S450L	Resistant	4.9 ± 2.3	49	4	13
	I491F	Resistant	5.1 ± 2.3	22	3	5
DNAG	S95T	Susceptible	−1.6 ± 1.8	50	5	15
	A90S	Hyper‐susceptible	−1.1 ± 1.4	33	5	8
	A90V	Resistant	2.0 ± 1.6	44	4	15
	E501D^c^	Resistant	−2.0 ± 3.2	59	9	10
	D94G	Resistant	−2.8 ± 8.9	45	4	10

^a^ *N* is the total number of free energy calculations used to calculate the ΔΔG, excluding the *rpoB* restraints as their contributions were negligible (see Supplementary Information S1).

^b^ *N*_min_ and *N*_max_ list the minimum and maximum number of repeat calculations used for apo or drug‐bound de‐charging (Δ*G*_qoff_), van der Waals (Δ*G*_vdW_) or re‐charging (Δ*G*_qon_) transitions, respectively (Figure 2).

^c^ Indicates a *gyrB* mutation.

By starting each simulation from a different structural seed and discarding the first half of the alchemical free energy simulations and then applying statistics to the resulting values of Δ*G* we are assuming that they are independent. If true, then one would also expect the values to be normally distributed which would appear to be the case for most sets of Δ*G* values (Figure 5). Applying the Shapiro‐Wilks test of normality to the *rpoB* data confirms that, despite the small numbers of samples in some cases, the majority of Δ*G* values are indeed normally distributed with the exceptions of Δ*G*_qon_ for the apo leg of S428C and Δ*G*_qon_ for the drug bound leg of S450L. For two DNA gyrase mutations there was also evidence of non‐normality in the Δ*G*_vdW_ for the apo leg of *gyrA* S95T and Δ*G*_on_ for both the apo and drug bound leg of *gyrA* D94G.

To test how far our simulations are from normality, we extended four apo and five drug‐bound simulations underlying the most variable component (qon, Figure 1) of the most complex mutation, *gyrA* D94G, by an order of magnitude (from 0.5 to 5 ns). As assessed by the Shapiro‐Wilks, the resulting distributions of apo‐ and drug‐bound free energies were indeed normal after 5 ns of simulation (*p =* 0.92 and *p =* 0.16) but the distribution of results for the repeated calculations, and therefore the error, remain large (Figure S3).

As we used thermodynamic integration with the trapezium rule to calculate free energies, the accuracy and precision is dependent on the degree of curvature in the component free energy calculations.^35^, ^37^ More complex transitions, that is, those with a greater difference in starting and end states, are likely to have increased curvature especially at *λ* values close to the endpoints (0 and 1). We therefore investigated the curvature in individual qoff, vdW and qon free energy components for a simple susceptible mutation, *gyrA* S95T, which involves the growing in of a methyl‐group and no change in charge, and the most complex mutation, *gyrA* D94G, which involves a significant reduction in atom number and removal of a full negative charge (Figure S4). As expected, for *gyrA* S95T, there was little curvature observed for either qon and qoff transitions as there is no major charge change. The vdW transition for both apo and drug‐bound systems, where the key change occurs in growing in a methyl group, does show some gentle curvature through mid‐range values of *λ* (Figure S4a), and the error for this transition was larger than for qoff or qon transitions (Figure 4B). In contrast, for both apo and drug‐bound legs of the D94G mutation, there is a high degree of curvature towards *λ* = 1 in the vdW transition where the large side chain of aspartate is removed (Figure S4b). Unexpectedly there was little curvature observed in the qoff transition, where the main negative charge of the aspartate side chain is removed, and the transition contributed a large amount of error (Figure S4b). In general, the individual *λ* values are much larger in magnitude for the *gyrA* D94G mutation than S95T, which is not unexpected due to the magnitude of change, but may explain why there is larger error associated with the *gyrA* D94G prediction (Figure 3B).

The accuracy and precision of the free energy calculations will also be dependent on the convergence of the individual calculations. Convergence of the calculations can be judged by the amount of overlap between energy distributions from neighboring *λ* windows, with overlap of >1% being considered reliable.^38^ For the complex D94G mutation we found instances of zero overlap between neighboring *λ* windows for both the Δ*G*_vdW_ and Δ*G*_qon_ calculations and therefore these calculations were not converged (Table S1). This is not unsurprising given the use of very short calculations but will affect the accuracy of quantitative results and could affect the accuracy and confidence intervals for the overall qualitative prediction. Conversely, for the less complex DNAG mutations there was no issues with the overlap (overlap data for S95T and the resistance conferring mutation A90V are shown in Table S1, data for A90S and E501D not shown).

## 4 DISCUSSION

We have shown how relative binding free energy (RBFE) techniques can be applied to large protein complexes to predict, with some success, the effect of individual protein mutations on the binding of an antibiotic, and thence whether resistance is conferred. When the size of the signal is large and the mutations do not involve significant changes in the electrical charge, as is the case for the *rpoB* mutations, one can successfully predict whether a mutation confers resistance to the antibiotic (in this case rifampicin). If the fold increase in minimum inhibitory concentration is small and/or there are significant charge changes, as is the case for most of the resistance conferring mutations in the DNA gyrase, then the estimated error of ΔΔ*G* will likely be so large that no definite prediction can be made. In addition, the observed non‐normality and lack of overlap between *λ* windows for constituent free energies for *gyrA* D94G indicates that these values are also not independent and not converged: to solve this either the *λ* simulations would have to be extended or the equilibration simulations would need to be more numerous as well as longer.

Despite the focus on resistance, it is more useful to be able to accurately and reproducibly predict susceptibility since clinically that will lead to immediate action, that is, starting the patient on the appropriate treatment regimen. A prediction of resistance will likely result in the sample being sent for further testing, at which point any incorrect predictions (false positives) will be detected. Due to the magnitudes of the errors and the fact that we are constrained to start from the wild‐type structure, it may be more difficult, unfortunately, to predict susceptibility than resistance using RBFE. For *rpoB* only one susceptible mutation could be confidently predicted. If we assume that most susceptible mutations will not affect the binding affinity for the antibiotic, then they would have a ΔΔG of zero. The predicted ΔΔ*G* of such mutations would hence require the estimated error to be at least less than the value of the ECOFF (for *rpoB* and DNA gyrase 1.2 and 0.9 kcal mol^−1^, respectively) to make a confident susceptible prediction. The magnitude of error for the mutations in this study was greater than the relevant ECOFF in all but one case (*rpoB* S388L, Table 1), and previous studies have found the typical error associated with RBFE calculations to be in the range of 1 kcal mol^−1.^ ^16^, ^17^, ^18^ It is likely that the error could be reduced by running a greater number of repeats, however some mutations can result in a small increase in MIC but not enough to confer resistance (as susceptibility can be defined as any MIC up to the ECOFF), and in such cases even a lower level of error (±0.5 kcal mol^−1^) may still prove insufficient for prediction.

Whilst alchemical binding free energy calculations therefore can play a role in predicting antibiotic resistance, the method may be most applicable when the target protein is small, non‐complex, and the magnitude of the change in the binding free energy large, as is the case for *S. aureus* DHFR and trimethoprim.^21^ For this system it has also been shown that it is possible to reduce the length of the simulations yet further but still maintain an accurate qualitative prediction.^25^ Taking all this together, we appear to have probed the limits (for now at least) of using RBFE methods to predict antibiotic resistance de novo. Interestingly, unlike most other applications of RBFE, one can tolerate large, estimated errors since we are ultimately only interested in the final binary classification of resistant or susceptible. A second and related application for RBFE is reducing the likelihood of a lead compound developing resistance by providing information during the development process of the likely mutations that could confer resistance and we hope to explore this in future work.

There are several shortcomings with our approach. We have assumed that resistance arises by reducing how well the antibiotic binds; this will not always be true. Secondly, our predictions depend on the accuracy of the molecular forcefields that describe the interatomic interactions; whilst the forcefield would be expected to be accurate for amino acids, which are the part of the system changing in the alchemical simulations, forcefields are not optimized for small molecules and ions^20^ and therefore may not be accurate. Indeed, in this study we needed to use a restraints on drug‐coordinated Mg^2+^ ions. Finally, we assume that the conformations used to seed each calculation are independent of one another and/or that the *λ* simulations are long enough to allow the initial state to be “forgotten”. Given we chose to use very short *λ* simulations the latter is almost certainly not true and whilst the majority of our calculated Δ*G* values appear to be normally distributed, some are not which is concerning. One would expect to have to run 4× the number of simulations to reduce the estimated error to half its original value if the simulations are independent. This makes simulations of these size prohibitively computationally intensive using the software and compute that we employed. Significant speed up could however be achieved by use of updated GROMACS software, where yearly updates have been shown to increase ns/day performance on a range of computational resource.^39^, ^40^

It is not in doubt that how the structure and dynamics of a protein change upon mutation contains valuable information that can, in theory, be used to predict whether individual mutations confer antibiotic resistance. Although RBFE may not (yet) be an appropriate tool for resistance prediction in the two complex systems we studied, several alternative routes exist. For instance, machine learning algorithms or energy scoring software such as Rosetta can predict binding free energies and these methods are less computationally intensive.^22^, ^41^, ^42^ Ultimately a combination of RBFE/MD and other approaches may not only complement one another but also form part of a larger toolkit that helps us to tackle antimicrobial resistance by improving diagnosis.

## 5 CONCLUSIONS

1. RBFE techniques can be applied to large protein complexes to predict resistance when the fold increase in minimum inhibitory concentration is large, and the mutations do not involve significant changes in the electrical charge.
2. RBFE methods may struggle to make predictions for large protein complexes with complex interactions such as these if the fold increase in minimum inhibitory concentration is small, due to the magnitude of the estimated errors.

## AUTHOR CONTRIBUTIONS

Alice E. Brankin and Philip W. Fowler designed the study, setup, ran, and analyzed the simulations and wrote the manuscript.

## Supporting information


**Figure S1** Bar chart showing effect on RFBE predictions when discarding different amounts of data from the start of 500 ps component FE calculations for (a) *gyrA* S95T and (b) *gyrA* D94G.
**Figure S2**. RBFE calculated mean ΔΔ*G* measurements of (a) rifampicin and (b) moxifloxacin resistance conferring mutations compared to the expected ΔΔ*G* measurement. Expected ΔΔ*G* measurements for each mutation were calculated from the geometric mean minimum inhibitory concentration (MIC) of a population of isolates containing each resistance conferring mutation and no other RNAP/DNAG mutation in an otherwise genetically wild‐type background, using previously described methods.^1^ Error bars represent 95% confidence interval, dotted lines represent the epidemiological cut off value (ECOFF) used to determine resistance and susceptibility.
**Figure S3**. Swarm plots of individual results from apo and drug bound 5 ns alchemical free energy calculations for the qon transition of DNAG *gyrA* D94G mutation. Results are normalized to the mean of the calculations for the apo leg. *p* values from Shapiro Wilks test are displayed.
**Figure S4**. Curvature in qoff, vdW and qon *λ*0 → 1 free energy calculations for (a) *gyrA* S95T and (b) *gyrA* D94G. Apo results are shown in light gray and drug‐bound results in dark gray.
**Table S1**. Percentage overlap between neighboring *λ* windows for qoff, vdW, qon transitions of apo and moxifloxacin bound legs of gyrA D94G, S95T and A90V mutations. All *λ* windows are evenly spaced.

gyrase_summary

rpob_summary

## Data Availability

The authors confirm that the data supporting the findings of this study are available within the article and its supplementary materials.

## References

[1] World Health Organization (WHO) , Rapid communication: Key changes to treatment of multidrug‐ and rifampicin‐resistant tuberculosis (MDR/RR‐TB) 2018.

[2] World Health Organization (WHO) , Catalogue of mutations in *Mycobacterium tuberculosis* complex and their association with drug resistance. License: CC BY‐NC‐SA 3.0 IGO. 2021.

[3] T. M. Walker , P. Miotto , C. U. Köser , P. W. Fowler , J. Knaggs , Z. Iqbal , M. Hunt , L. Chindelevitch , M. R. Farhat , D. M. Cirillo , I. Comas , J. Posey , S. V. Omar , T. E. A. Peto , A. Suresh , S. Uplekar , S. Laurent , R. E. Colman , C.‐M. Nathanson , M. Zignol , A. S. Walker , D. W. Crook , N. Ismail , T. C. Rodwell , A. S. Walker , A. J. C. Steyn , A. Lalvani , A. Baulard , A. Christoffels , A. Mendoza‐Ticona , A. Trovato , A. Skrahina , A. S. Lachapelle , A. Brankin , A. Piatek , A. Gibertoni Cruz , A. Koch , A. M. Cabibbe , A. Spitaleri , A. P. Brandao , A. Chaiprasert , A. Suresh , A. Barbova , A. Van Rie , A. Ghodousi , A. Bainomugisa , A. Mandal , A. Roohi , B. Javid , B. Zhu , B. Letcher , C. Rodrigues , C. Nimmo , C.‐M. Nathanson , C. Duncan , C. Coulter , C. Utpatel , C. Liu , C. Grazian , C. Kong , C. U. Köser , D. J. Wilson , D. M. Cirillo , D. Matias , D. Jorgensen , D. Zimenkov , D. Chetty , D. A. J. Moore , D. A. Clifton , D. W. Crook , D. van Soolingen , D. Liu , D. Kohlerschmidt , D. Barreira , D. Ngcamu , E. D. Santos Lazaro , E. Kelly , E. Borroni , E. Roycroft , E. Andre , E. C. Böttger , E. Robinson , F. Menardo , F. F. Mendes , F. B. Jamieson , F. Coll , G. F. Gao , G. W. Kasule , G. M. Rossolini , G. Rodger , E. G. Smith , G. Meintjes , G. Thwaites , H. Hoffmann , H. Albert , H. Cox , I. F. Laurenson , I. Comas , I. Arandjelovic , I. Barilar , J. Robledo , J. Millard , J. Johnston , J. Posey , J. R. Andrews , J. Knaggs , J. Gardy , J. Guthrie , J. Taylor , J. Werngren , J. Metcalfe , J. Coronel , J. Shea , J. Carter , J. M. W. Pinhata , J. V. Kus , K. Todt , K. Holt , K. S. Nilgiriwala , K. T. Ghisi , K. M. Malone , K. Faksri , K. A. Musser , L. Joseph , L. Rigouts , L. Chindelevitch , L. Jarrett , L. Grandjean , L. Ferrazoli , M. Rodrigues , M. Farhat , M. Schito , M. M. Fitzgibbon , M. M. Loembé , M. Wijkander , M. Ballif , M.‐S. Rabodoarivelo , M. Mihalic , M. Wilcox , M. Hunt , M. Zignol , M. Merker , M. Egger , M. O'Donnell , M. Caws , M.‐H. Wu , M. G. Whitfield , M. Inouye , M. Mansjö , M. H. Dang Thi , M. Joloba , S. M. M. Kamal , N. Okozi , N. Ismail , N. Mistry , N. N. Hoang , N. Rakotosamimanana , N. I. Paton , P. M. V. Rancoita , P. Miotto , P. Lapierre , P. J. Hall , P. Tang , P. Claxton , P. Wintringer , P. M. Keller , P. V. K. Thai , P. W. Fowler , P. Supply , P. Srilohasin , P. Suriyaphol , P. Rathod , P. Kambli , R. Groenheit , R. E. Colman , R. T.‐H. Ong , R. M. Warren , R. J. Wilkinson , R. Diel , R. S. Oliveira , R. Khot , R. Jou , S. Tahseen , S. Laurent , S. Gharbia , S. Kouchaki , S. Shah , S. Plesnik , S. G. Earle , S. Dunstan , S. J. Hoosdally , S. Mitarai , S. Gagneux , S. V. Omar , S.‐Y. Yao , S. Grandjean Lapierre , S. Battaglia , S. Niemann , S. Pandey , S. Uplekar , T. A. Halse , T. Cohen , T. Cortes , T. Prammananan , T. A. Kohl , N. T. T. Thuong , T. Y. Teo , T. E. A. Peto , T. C. Rodwell , T. William , T. M. Walker , T. R. Rogers , U. Surve , V. Mathys , V. Furió , V. Cook , S. Vijay , V. Escuyer , V. Dreyer , V. Sintchenko , V. Saphonn , W. Solano , W.‐H. Lin , W. van Gemert , W. He , Y. Yang , Y. Zhao , Y. Qin , Y.‐X. Xiao , Z. Hasan , Z. Iqbal , Z. M. Puyen , Lancet Microbe 2022, 3(4), e265. 35373160 10.1016/S2666-5247(21)00301-3 PMC7612554

[4] A. Brankin , K. M. Malone , I. Barilar , S. Battaglia , E. Borroni , A. P. Brandao , A. M. Cabibbe , J. Carter , D. M. Cirillo , P. Claxton , D. A. Clifton , T. Cohen , J. Coronel , D. W. Crook , V. Dreyer , S. G. Earle , V. Escuyer , L. Ferrazoli , P. W. Fowler , G. F. Gao , J. Gardy , S. Gharbia , K. T. Ghisi , A. Ghodousi , A. L. G. Cruz , L. Grandjean , C. Grazian , R. Groenheit , J. L. Guthrie , W. He , H. Hoffmann , S. J. Hoosdally , M. Hunt , Z. Iqbal , N. A. Ismail , L. Jarrett , L. Joseph , R. Jou , P. Kambli , R. Khot , J. Knaggs , A. Koch , D. Kohlerschmidt , S. Kouchaki , A. S. Lachapelle , A. Lalvani , S. G. Lapierre , I. F. Laurenson , B. Letcher , W.‐H. Lin , C. Liu , D. Liu , A. Mandal , M. Mansjö , D. Matias , G. Meintjes , F. de Freitas Mendes , M. Merker , M. Mihalic , J. Millard , P. Miotto , N. Mistry , D. Moore , K. A. Musser , D. Ngcamu , H. N. Nhung , S. Niemann , K. S. Nilgiriwala , C. Nimmo , N. Okozi , R. S. Oliveira , S. V. Omar , N. Paton , T. E. Peto , J. M. W. Pinhata , S. Plesnik , Z. M. Puyen , M. S. Rabodoarivelo , N. Rakotosamimanana , P. M. Rancoita , P. Rathod , E. Robinson , G. Rodger , C. Rodrigues , T. C. Rodwell , A. Roohi , D. Santos‐Lazaro , S. Shah , T. A. Kohl , G. Smith , W. Solano , A. Spitaleri , P. Supply , U. Surve , S. Tahseen , N. T. T. Thuong , G. Thwaites , K. Todt , A. Trovato , C. Utpatel , A. Van Rie , S. Vijay , T. M. Walker , A. Sarah Walker , R. Warren , J. Werngren , M. Wijkander , R. J. Wilkinson , D. J. Wilson , P. Wintringer , Y.‐X. Xiao , Y. Yang , Z. Yanlin , S.‐Y. Yao , B. Zhu , PLOS Biology 2022, 20(8), e3001721. 35944069 10.1371/journal.pbio.3001721 PMC9363010

[5] The CRyPTIC Consortium , C. Allix‐Beguec , I. Arandjelovic , L. Bi , P. Beckert , M. Bonnet , P. Bradley , A. M. Cabibbe , I. Cancino‐Munoz , M. J. Caulfield , A. Chaiprasert , D. M. Cirillo , D. A. Clifton , I. Comas , D. W. Crook , M. R. De Filippo , H. de Neeling , R. Diel , F. A. Drobniewski , K. Faksri , M. R. Farhat , J. Fleming , P. Fowler , T. A. Fowler , Q. Gao , J. Gardy , D. Gascoyne‐Binzi , A. L. Gibertoni‐Cruz , A. Gil‐Brusola , T. Golubchik , X. Gonzalo , L. Grandjean , G. He , J. L. Guthrie , S. Hoosdally , M. Hunt , Z. Iqbal , N. Ismail , J. Johnston , F. M. Khanzada , C. C. Khor , T. A. Kohl , C. Kong , S. Lipworth , Q. Liu , G. Maphalala , E. Martinez , V. Mathys , M. Merker , P. Miotto , N. Mistry , D. A. J. Moore , M. Murray , S. Niemann , S. V. Omar , R. T. Ong , T. E. A. Peto , J. E. Posey , T. Prammananan , A. Pym , C. Rodrigues , M. Rodrigues , T. Rodwell , G. M. Rossolini , E. Sanchez Padilla , M. Schito , X. Shen , J. Shendure , V. Sintchenko , A. Sloutsky , E. G. Smith , M. Snyder , K. Soetaert , A. M. Starks , P. Supply , P. Suriyapol , S. Tahseen , P. Tang , Y. Y. Teo , T. N. T. Thuong , G. Thwaites , E. Tortoli , D. van Soolingen , A. S. Walker , T. M. Walker , M. Wilcox , D. J. Wilson , D. Wyllie , Y. Yang , H. Zhang , Y. Zhao , B. Zhu , N. Engl. J. Med. 2018, 379(15), 1403. 30280646 10.1056/NEJMoa1800474 PMC6121966

[6] C. C. Boehme , P. Nabeta , D. Hillemann , M. P. Nicol , S. Shenai , F. Krapp , J. Allen , R. Tahirli , R. Blakemore , R. Rustomjee , A. Milovic , M. Jones , S. M. O'Brien , D. H. Persing , S. Ruesch‐Gerdes , E. Gotuzzo , C. Rodrigues , D. Alland , M. D. Perkins , N. Engl. J. Med. 2010, 363(11), 1005. 20825313 10.1056/NEJMoa0907847 PMC2947799

[7] World Health Organization (WHO) . WHO policy statement: Automated real‐time nucleic acid amplification technology for rapid and simultaneous detection of tuberculosis and rifampicin resistance: Xpert MTB/RIF system 2011, Accessed July 24, 2014. http://whqlibdoc.who.int/publications/2011/9789241501545_eng.pdf. 26158191

[8] E. Sanchez‐Padilla , M. Merker , P. Beckert , F. Jochims , T. Dlamini , P. Kahn , M. Bonnet , S. Niemann , N. Engl. J. Med. 2015, 372(12), 1181. 25785984 10.1056/NEJMc1413930

[9] M. R. Farhat , K. R. Jacobson , M. F. Franke , D. Kaur , A. Sloutsky , C. D. Mitnick , M. Murray , J. Clin. Microbiol. 2016, 54(3), 727. 26763957 10.1128/JCM.02775-15 PMC4767988

[10] P. Miotto , B. Tessema , E. Tagliani , L. Chindelevitch , A. M. Starks , C. Emerson , D. Hanna , P. S. Kim , R. Liwski , M. Zignol , C. Gilpin , S. Niemann , C. M. Denkinger , J. Fleming , R. M. Warren , D. Crook , J. Posey , S. Gagneux , S. Hoffner , C. Rodrigues , I. Comas , D. M. Engelthaler , M. Murray , D. Alland , L. Rigouts , C. Lange , K. Dheda , R. Hasan , U. D. K. Ranganathan , R. McNerney , M. Ezewudo , D. M. Cirillo , M. Schito , C. U. Koser , T. C. Rodwell , Eur. Respir. J. 2017, 50(6), 1701354. 29284687 10.1183/13993003.01354-2017 PMC5898944

[11] T. M. Walker , T. A. Kohl , S. V. Omar , J. Hedge , C. Del Ojo Elias , P. Bradley , Z. Iqbal , S. Feuerriegel , K. E. Niehaus , D. J. Wilson , D. A. Clifton , G. Kapatai , C. L. C. Ip , R. Bowden , F. A. Drobniewski , C. Allix‐Beguec , C. Gaudin , J. Parkhill , R. Diel , P. Supply , D. W. Crook , E. G. Smith , A. S. Walker , N. Ismail , S. Niemann , T. E. A. Peto , Lancet Infect. Dis. 2015, 15(10), 1193. 26116186 10.1016/S1473-3099(15)00062-6 PMC4579482

[12] The CRyPTIC Consortium , J. J. Carter , bioRxiv 2021. 10.1101/2021.09.14.460353

[13] H. E. Takiff , L. Salazar , C. Guerrero , W. Philipp , W. M. Huang , B. Kreiswirth , S. T. Cole , W. R. Jacobs Jr. , A. Telenti , Antimicrob. Agents Chemother. 1994, 38(4), 773. 8031045 10.1128/aac.38.4.773 PMC284541

[14] A. Ajileye , N. Alvarez , M. Merker , T. M. Walker , S. Akter , K. Brown , D. Moradigaravand , T. Schon , S. Andres , V. Schleusener , S. V. Omar , F. Coll , H. Huang , R. Diel , N. Ismail , J. Parkhill , B. C. de Jong , T. E. Peto , D. W. Crook , S. Niemann , J. Robledo , E. G. Smith , S. J. Peacock , C. U. Koser , Antimicrob. Agents Chemother. 2017, 61(4), e02169. 28137812 10.1128/AAC.02169-16 PMC5365657

[15] A. Pantel , S. Petrella , N. Veziris , F. Brossier , S. Bastian , V. Jarlier , C. Mayer , A. Aubry , Antimicrob. Agents Chemother. 2012, 56(4), 1990. 22290942 10.1128/AAC.06272-11 PMC3318379

[16] L. Wang , Y. Wu , Y. Deng , B. Kim , L. Pierce , G. Krilov , D. Lupyan , S. Robinson , M. K. Dahlgren , J. Greenwood , D. L. Romero , C. Masse , J. L. Knight , T. Steinbrecher , T. Beuming , W. Damm , E. Harder , W. Sherman , M. Brewer , R. Wester , M. Murcko , L. Frye , R. Farid , T. Lin , D. L. Mobley , W. L. Jorgensen , B. J. Berne , R. A. Friesner , R. Abel , J. Am. Chem. Soc. 2015, 137(7), 2695. 25625324 10.1021/ja512751q

[17] V. Gapsys , L. Pérez‐Benito , M. Aldeghi , D. Seeliger , H. van Vlijmen , G. Tresadern , B. L. de Groot , Chem. Sci. 2020, 11(4), 1140. 10.1039/c9sc03754c PMC8145179 34084371

[18] T. B. Steinbrecher , M. Dahlgren , D. Cappel , T. Lin , L. Wang , G. Krilov , R. Abel , R. Friesner , W. Sherman , J. Chem. Inf. Model. 2015, 55(11), 2411. 26457994 10.1021/acs.jcim.5b00538

[19] J. W. Ponder , D. A. Case , Adv. Protein Chem. 2003, 66, 27. 14631816 10.1016/s0065-3233(03)66002-x

[20] M. Lundborg , E. Lindahl , J. Phys. Chem. B 2015, 119(3), 810. 25343332 10.1021/jp505332p

[21] P. W. Fowler , K. Cole , N. C. Gordon , A. M. Kearns , M. J. Llewelyn , T. E. A. Peto , D. W. Crook , A. S. Walker , Cell Chem. Biol. 2018, 25(3), 339. 29307840 10.1016/j.chembiol.2017.12.009

[22] K. Hauser , C. Negron , S. K. Albanese , S. Ray , T. Steinbrecher , R. Abel , J. D. Chodera , L. Wang , Commun. Biol. 2018, 1, 70. 30159405 10.1038/s42003-018-0075-x PMC6110136

[23] M. Aldeghi , V. Gapsys , B. L. de Groot , ACS Cent. Sci. 2019, 5(8), 1468. 31482130 10.1021/acscentsci.9b00590 PMC6716344

[24] A. P. Bhati , S. Wan , P. V. Coveney , J. Chem. Theory Comput. 2019, 15(2), 1265. 30592603 10.1021/acs.jctc.8b01118 PMC6447239

[25] P. W. Fowler , Interf. Focus 2020, 10(6), 20190141. 10.1098/rsfs.2019.0141 PMC7653339 33178416

[26] World Health Organization (WHO) , Technical report on critical concentrations for drug susceptibility testing of isoniazid and the rifamycins (rifampicin, rifabutin and rifapentine), 2021.

[27] T. R. Blower , B. H. Williamson , R. J. Kerns , J. M. Berger , Proc. Natl. Acad. Sci. U. S. A. 2016, 113(7), 1706. 26792525 10.1073/pnas.1525047113 PMC4763791

[28] W. Lin , S. Mandal , D. Degen , Y. Liu , Y. W. Ebright , S. Li , Y. Feng , Y. Zhang , S. Mandal , Y. Jiang , S. Liu , M. Gigliotti , M. Talaue , N. Connell , K. Das , E. Arnold , R. H. Ebright , Mol. Cell 2017, 66(2), 169 e8. 28392175 10.1016/j.molcel.2017.03.001 PMC5438085

[29] A. Fiser , A. Sali , Bioinformatics 2003, 19(18), 2500. 14668246 10.1093/bioinformatics/btg362

[30] K. Lindorff‐Larsen , S. Piana , K. Palmo , P. Maragakis , J. L. Klepeis , R. O. Dror , D. E. Shaw , Proteins 2010, 78(8), 1950. 20408171 10.1002/prot.22711 PMC2970904

[31] T. M. Mark James Abraham , R. Schulz , S. Páll , J. C. Smith , B. Hess , E. Lindahl , SoftwareX 2015, 1‐2, 19.

[32] B. Hess , H. Bekker , H. J. C. Berendsen , J. G. E. M. Fraaije , J. Comput. Chem. 1997, 18(12), 1463.

[33] V. Gapsys , S. Michielssens , D. Seeliger , B. L. de Groot , J. Comput. Chem. 2015, 36(5), 348. 25487359 10.1002/jcc.23804 PMC4365728

[34] E. Jefferys , Z. A. Sands , J. Shi , M. S. Sansom , P. W. Fowler , J. Chem. Theory Comput. 2015, 11(6), 2743. 26089745 10.1021/ct501111d PMC4467903

[35] A. S. J. S. Mey , B. K. Allen , H. E. Bruce McDonald , J. D. Chodera , D. F. Hahn , M. Kuhn , J. Michel , D. L. Mobley , L. N. Naden , S. Prasad , A. Rizzi , J. Scheen , M. R. Shirts , G. Tresadern , H. Xu , Liv. J. Comput. Mol. Sci. 2020, 2(1), 18378. 10.33011/livecoms.2.1.18378 PMC8388617 34458687

[36] Consortium, T. C , Eur. Respir. J. 2022, 60(2), 2200239.

[37] T. T. Pham , M. R. Shirts , J. Chem. Phys. 2011, 135(3), 034114. 21786994 10.1063/1.3607597

[38] G. König , B. R. Brooks , W. Thiel , D. M. York , Mol. Simul. 2018, 44(13–14), 1062. 30581251 10.1080/08927022.2018.1475741 PMC6298030

[39] C. Kutzner , C. Kniep , A. Cherian , L. Nordstrom , H. Grubmüller , B. L. de Groot , V. Gapsys , J. Chem. Inf. Model. 2022, 62, 1691. 35353508 10.1021/acs.jcim.2c00044 PMC9006219

[40] C. Kutzner , S. Páll , M. Fechner , A. Esztermann , B. L. de Groot , H. Grubmüller , J. Comput. Chem. 2019, 40(27), 2418. 31260119 10.1002/jcc.26011

[41] D. E. Pires , D. B. Ascher , T. L. Blundell , Bioinformatics 2014, 30(3), 335. 24281696 10.1093/bioinformatics/btt691 PMC3904523

[42] M. Aldeghi , V. Gapsys , B. L. de Groot , ACS Cent. Sci. 2018, 4(12), 1708. 30648154 10.1021/acscentsci.8b00717 PMC6311686

