# Supplementary material for: Predicting antibiotic resistance in complex protein targets using alchemical free energy methods
Source: J Comput Chem. 2022 Aug 25;43(26):1771–82. doi: 10.1002/jcc.26979 (PMC9545121; doi:10.1002/jcc.26979)
Supplement: Supplementary file 1 — Figure S1 Bar chart showing effect on RFBE predictions when discarding different amounts of data from the start of 500 ps component FE calculations for (a) gyrA S95T and (b) gyrA D94G. Figure S2. RBFE calculated mean ΔΔG measurements of (a) rifampicin and (b) moxifloxacin resistance conferring mutations compared to the expected ΔΔG measurement. Expected ΔΔG measurements for each mutation were calculated from the geometric mean minimum inhibitory concentration (MIC) of a population of isolates containing each resistance conferring mutation and no other RNAP/DNAG mutation in an otherwise genetically wild‐type background, using previously described methods. 1 Error bars represent 95% confidence interval, dotted lines represent the epidemiological cut off value (ECOFF) used to determine resistance and susceptibility. Figure S3. Swarm plots of individual results from apo and drug bound 5 ns alchemical free energy calculations for the qon transition of DNAG gyrA D94G mutation. Results are normalized to the mean of the calculations for the apo leg. p values from Shapiro Wilks test are displayed. Figure S4. Curvature in qoff, vdW and qon λ0 → 1 free energy calculations for (a) gyrA S95T and (b) gyrA D94G. Apo results are shown in light gray and drug‐bound results in dark gray. Table S1. Percentage overlap between neighboring λ windows for qoff, vdW, qon transitions of apo and moxifloxacin bound legs of gyrA D94G, S95T and A90V mutations. All λ windows are evenly spaced. [file JCC-43-1771-s003.docx]

Predicting antibiotic resistance in complex protein targets using alchemical free energy methods – Supplementary Figures and Tables

Alice E Brankin^1^ and Philip W Fowler^1,2^*

1. Nuffield Department of Medicine, John Radcliffe Hospital, University of Oxford, Oxford OX3 9DU, UK
2. National Institute of Health Research Oxford Biomedical Research Centre, John Radcliffe Hospital, Headley Way, Oxford OX3 9DU, UK


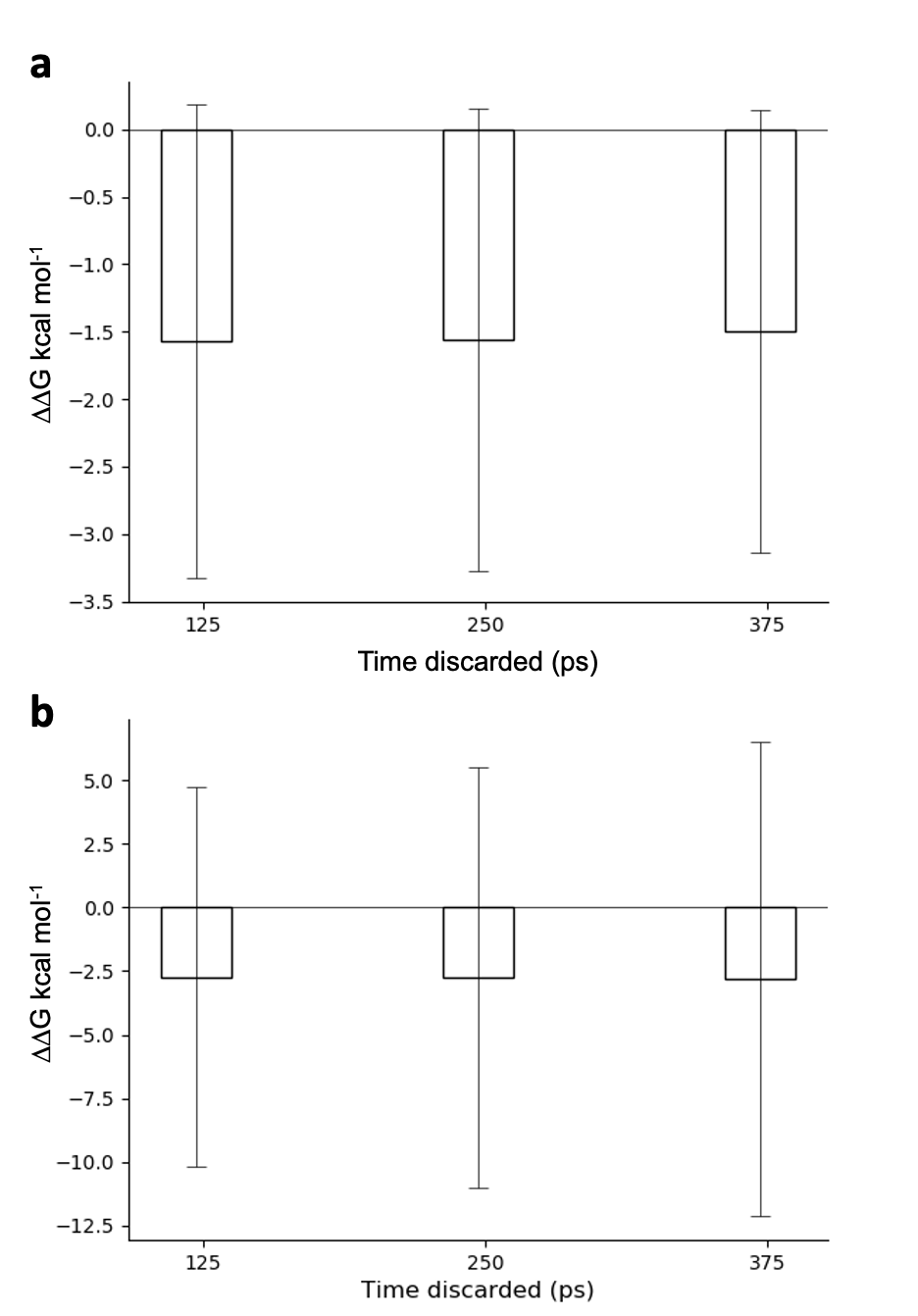


**Figure S1.** Bar chart showing effect on RFBE predictions when discarding different amounts of data from the start of 500 ps component FE calculations for a) gyrA S95T and b) gyrA D94G


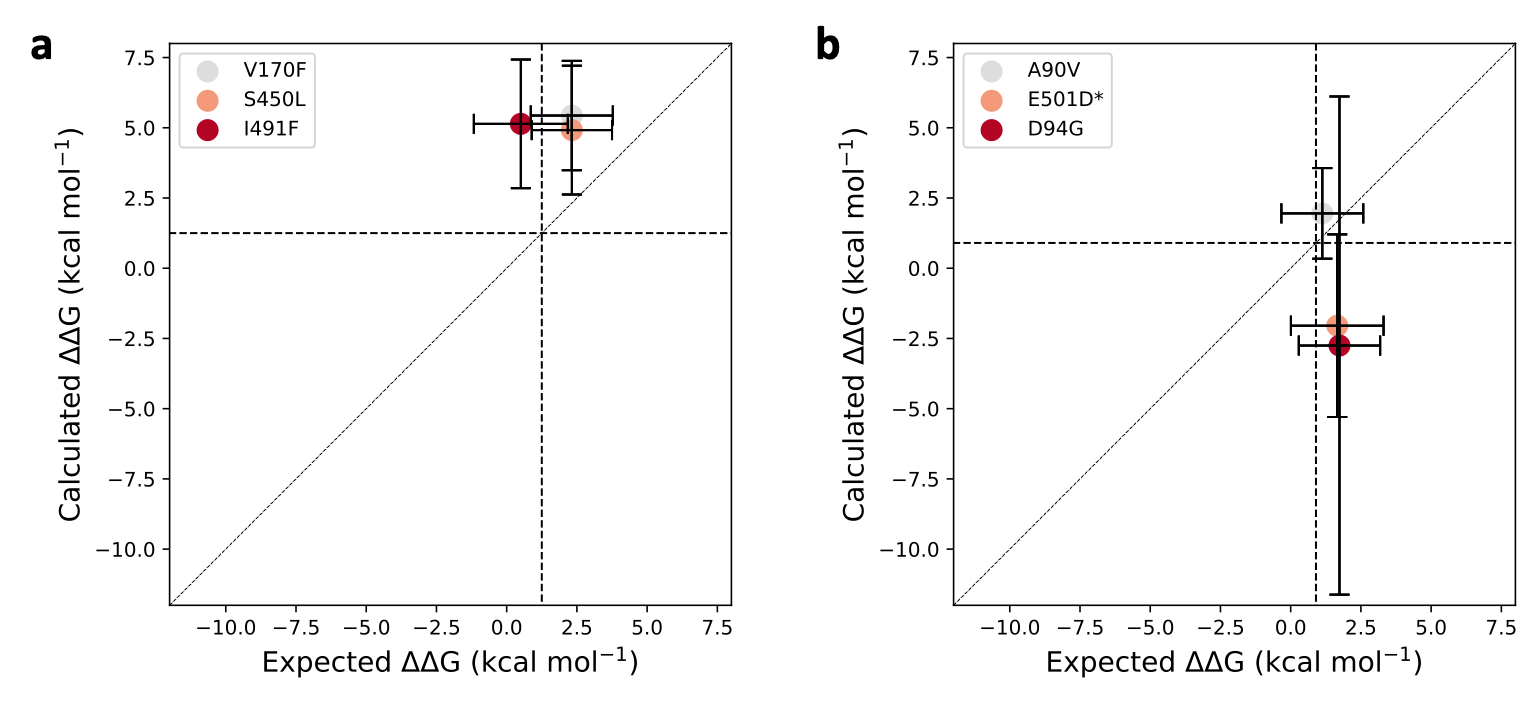


**Figure S2.** RBFE calculated mean ΔΔG measurements of (a) rifampicin and (b) moxifloxacin resistance conferring mutations compared to the expected ΔΔG measurement. Expected ΔΔG measurements for each mutation were calculated from the geometric mean minimum inhibitory concentration (MIC) of a population of isolates containing each resistance conferring mutation and no other RNAP/DNAG mutation in an otherwise genetically wild-type background, using previously described methods^1^. Error bars represent 95% confidence interval, dotted lines represent the epidemiological cut off value (ECOFF) used to determine resistance and susceptibility.

**Figure S3.** Swarm plots of individual results from apo and drug bound 5 ns alchemical free energy calculations for the qon transition of DNA gyrase gyrA D94G mutation. Results are normalised to the mean of the calculations for the apo leg. p values from Shapiro Wilks test are displayed.


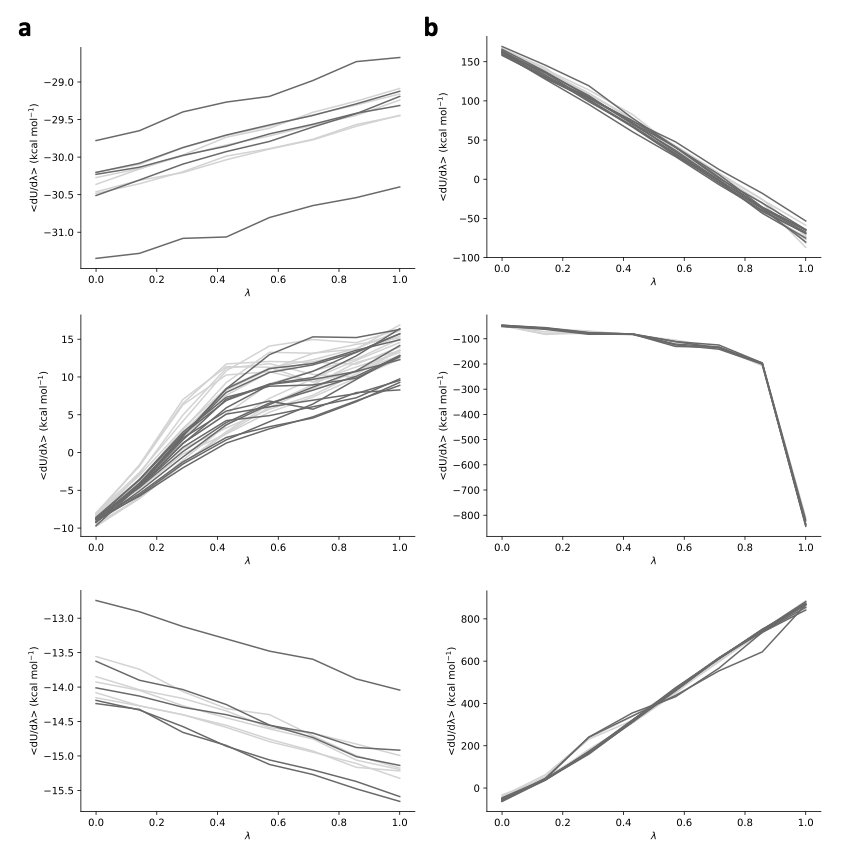


**Figure S4.** Curvature in qoff, vdW and qon λ0→1 free energy calculations for (a) gyrA S95T and (b) gyrA D94G. Apo results are shown in light grey and drug-bound results in dark grey.

**Table S1.** Percentage overlap between neighboring λ windows for qoff, vdW, qon transitions of apo and moxifloxacin bound legs of gyrA D94G, S95T and A90V mutations. All λ windows are evenly spaced.

|  |  |  | **λ window overlap (%)** | | | | | | |
| --- | --- | --- | --- | --- | --- | --- | --- | --- | --- |
|  |  |  | **0-1** | **1-2** | **2-3** | **3-4** | **4-5** | **5-6** | **6-7** |
| **D94G** | **mfx-bound** | **qoff** | 17.5 | 21.4 | 14.8 | 16.9 | 15.6 | 14.8 | 5.2 |
| **D94G** | **mfx-bound** | **vdW** | 68.7 | 52.4 | 90.0 | 43.5 | 47.6 | 16.4 | 0.0 |
| **D94G** | **mfx-bound** | **qon** | 1.2 | 0.0 | 1.5 | 1.6 | 0.8 | 1.9 | 0.0 |
| **D94G** | **apo** | **qoff** | 19.1 | 21.9 | 17.0 | 11.6 | 11.2 | 15.8 | 14.7 |
| **D94G** | **apo** | **vdW** | 74.7 | 74.2 | 62.9 | 46.4 | 53.9 | 8.9 | 0.0 |
| **D94G** | **apo** | **qon** | 3.8 | 0.8 | 0.1 | 0.3 | 0.1 | 0.0 | 1.3 |
| **S95T** | **mfx-bound** | **qoff** | 94.0 | 92.3 | 94.8 | 93.0 | 92.9 | 90.8 | 97.6 |
| **S95T** | **mfx-bound** | **vdW** | 14.6 | 27.9 | 57.1 | 85.8 | 93.6 | 90.0 | 85.8 |
| **S95T** | **mfx-bound** | **qon** | 95.3 | 91.4 | 93.5 | 90.6 | 94.5 | 92.1 | 95.6 |
| **S95T** | **apo** | **qoff** | 96.0 | 89.9 | 92.6 | 92.9 | 94.1 | 86.1 | 95.3 |
| **S95T** | **apo** | **vdW** | 21.1 | 28.9 | 56.2 | 72.2 | 83.7 | 82.5 | 89.6 |
| **S95T** | **apo** | **qon** | 94.5 | 91.0 | 93.2 | 94.3 | 92.5 | 95.1 | 90.4 |
| **A90V** | **mfx-bound** | **qoff** | 97.0 | 97.4 | 97.0 | 97.2 | 96.6 | 96.5 | 96.5 |
| **A90V** | **mfx-bound** | **vdW** | 4.6 | 10.4 | 37.1 | 65.0 | 75.7 | 82.2 | 78.4 |
| **A90V** | **mfx-bound** | **qon** | 97.7 | 91.5 | 96.7 | 92.6 | 90.2 | 95.2 | 94.7 |
| **A90V** | **apo** | **qoff** | 95.9 | 98.4 | 96.5 | 97.9 | 97.2 | 98.2 | 95.0 |
| **A90V** | **apo** | **vdW** | 4.6 | 5.8 | 45.7 | 85.6 | 92.0 | 79.7 | 73.3 |
| **A90V** | **apo** | **qon** | 93.5 | 94.6 | 94.2 | 91.5 | 95.0 | 93.2 | 95.1 |

**References**

1. Fowler, P. W.; Cole, K.; Gordon, N. C.; Kearns, A. M.; Llewelyn, M. J.; Peto, T. E. A.; Crook, D. W.; Walker, A. S., Robust Prediction of Resistance to Trimethoprim in Staphylococcus aureus. *Cell Chem Biol* **2018,** *25* (3), 339-349 e4.
